# Capacitance Enhancement of Hydrothermally Reduced Graphene Oxide Nanofibers

**DOI:** 10.3390/nano10061056

**Published:** 2020-05-30

**Authors:** Daniel Torres, Sara Pérez-Rodríguez, David Sebastián, José Luis Pinilla, María Jesús Lázaro, Isabel Suelves

**Affiliations:** Instituto de Carboquímica, Consejo Superior de Investigaciones Científicas (CSIC), Miguel Luesma Castán 4, 50018 Zaragoza, Spain; sperez@icb.csic.es (S.P.-R.); dsebastian@icb.csic.es (D.S.); jlpinilla@icb.csic.es (J.L.P.); mlazaro@icb.csic.es (M.J.L.); isuelves@icb.csic.es (I.S.)

**Keywords:** carbon nanofibers, reduced graphene oxide nanofibers, hydrothermal reduction, capacitance

## Abstract

Nanocarbon materials present sp^2^-carbon domains skilled for electrochemical energy conversion or storage applications. In this work, we investigate graphene oxide nanofibers (GONFs) as a recent interesting carbon material class. This material combines the filamentous morphology of the starting carbon nanofibers (CNFs) and the interlayer spacing of graphene oxide, and exhibits a domain arrangement accessible for fast transport of electrons and ions. Reduced GONFs (RGONFs) present the partial removal of basal functional groups, resulting in higher mesoporosity, turbostratic stacking, and surface chemistry less restrictive for transport phenomena. Besides, the filament morphology minimizes the severe layer restacking shown in the reduction of conventional graphene oxide sheets. The influence of the reduction temperature (140–220 °C) on the electrochemical behaviour in aqueous 0.5 M H_2_SO_4_ of RGONFs is reported. RGONFs present an improved capacitance up to 16 times higher than GONFs, ascribed to the unique structure of RGONFs containing accessible turbostratic domains and restored electronic conductivity. Hydrothermal reduction at 140 °C results in the highest capacitance as evidenced by cyclic voltammetry and electrochemical impedance spectroscopy measurements (up to 137 F·g^−1^). Higher temperatures lead to the removal of sulphur groups and slightly thicker graphite domains, and consequently a decrease of the capacitance.

## 1. Introduction

Carbon nanofibers (CNFs), composed entirely of graphite stacks of small lateral size in a 1D filament morphology, present a unique structure that combines properties such as electronic conduction, thermal and chemical resistance, low mass density and good specific surface area, which are essential for a wide range of potential applications. In this sense, CNFs have been used for many different applications, such as heterogeneous catalysis, conversion or storage of electrochemical energy, sensors, electronic devices, and structural and conductive composite materials [1,2,3,4,5,6,7]. In the case of electrochemical energy storage, the high electrical conductivity of CNFs in combination with their accessible porosity allows satisfactory charge accumulation but lower than that offered for graphene materials [8,9]. Graphene is the basic structural unit of the most conductive nanocarbon materials including graphite and carbon nanofibers and nanotubes since it exhibits a free movement of π-electrons along with the 2D network of conjugated sp^2^-carbon, allowing it to achieve ultrahigh electrical conductivities (10^5^–10^6^ S·m^−1^) [7,10]. Graphene has already been used in all possible electrochemical devices with remarkable results [11], whereas expanded graphite presented a large volumetric capacitance (177 F·cm^−3^) in aqueous acid media [12]. Likewise, graphite intercalation compounds have even offered conductivities higher than metallic copper, showing notable performance in batteries motivated by rapid diffusion of species between its stacks of distanced graphene layers [13].

In this direction, graphene oxide (GO) has been tentatively investigated for energy conversion and storage applications but oxygen interrupts the free electron movement compromising the electrical conductivity of the graphene layers [14,15]. On the other hand, functionalization enhances the hydrophilicity of nanocarbon materials, which leads to an improved electrochemical capacitance in aqueous electrolytes. GO is conventionally obtained from graphite by chemical oxidation routes, which involve the use of a strong oxidant in acid medium, such as the Hummers method [16], which besides to the creation of oxygen functionalities, including in-plane (hydroxyl and epoxide groups) and edge groups (carbonyl and carboxylic groups) [17,18], results in the generation of sulphur-containing groups [19,20]. As a result of the oxidation, lattice defects (vacancies, holes) are generated in the graphene network which may alter the interaction of the carbon surface with the electrolyte, and thus the electrochemical behaviour.

Graphene oxide nanofibers (GONFs) arise as a new alternative for catalytic and electrochemical applications offering a combination of the best characteristics of CNFs and GO materials: filamentous configuration, accessible microporosity, and tuneable functionalization. Promising approaches in the recent literature related to the combination of GO and CNFs in nanocomposites [21,22,23] or CNFs produced from GO precursors by electrospinning [24], by templating procedures [25], or by controlled assembly [26], with interesting synergies within phases, have been reported. Only a few recent works are found to report the oxidation and exfoliation of CNFs as a procedure to result in GONFs [27,28]. The innovative aspect of this article is that GONFs are a result of the chemical exfoliation of CNFs at the surface level, representing a novel approach to the best of our knowledge. The main advantage is that the controlled oxidation of CNFs allows keeping the filament morphology. An extended oxidation of CNFs causes its downsizing to few-layer graphene sheets and graphene quantum dots [29].

In the present work, the hydrothermal reduction of GONFs and the effect of the temperature on their physicochemical and electrochemical properties are studied. Stoller and co-workers reported an enhancement of the electrochemical behaviour of GO materials by removal of the large amounts of oxygen groups that diminish the electrical conductivity and hinder the access of species through its porosity [30]. However, oxygen removal gives place to the severe and irreversible restacking of individual graphene sheets (higher *L*_c_ and number of layers per stack), which decreases the specific capacitance of RGO (reduced GO) with respect to the expected theoretical values for graphene (550 F·g^−1^) [31]. In that sense, layer restacking causes that a large surface area of RGO becomes inaccessible for charge storage [32]. In case of processing reduced GONFs (RGONFs), the filamentous arrangement of GO stacks largely inhibits this restacking (stacks with a number of layers below 10), offering the possibility of obtaining carbon nanofibers with domains of turbostratic graphite, structure more accessible for fast transport of electrons and reactants than hexagonal graphite. Likewise, particles of nanofilaments simulate the inverse structure of a porous support preserving mesoporosity, which prevents any mass or electron transfer limitations [33]. Finally, the partial reduction of this material barely alters its hydrophilicity, so important for electrochemical applications in aqueous media [34]. The electrochemical capacitance and the electrode/electrolyte interface of the resultant RGONFs are analysed by cyclic voltammetry and impedance electrochemical spectroscopy in acid medium (0.5 M H_2_SO_4_). Herein, the electrochemical properties of RGONFs are studied for the first time and correlated to their physicochemical features.

## 2. Materials and Methods

### 2.1. Preparation of Fishbone GONFs and RGONFs

GONFs were obtained by chemical oxidation, using the modified Hummers method [16,35,36], and ultrasound-assisted exfoliation of fishbone CNFs according to our optimized method [29], where the synthesis method of the starting CNFs is also described. Briefly, 3.0 g of CNFs, 3.0 g of NaNO_3_ (99%), and 138 mL of H_2_SO_4_ (96%) were mixed in an ice bath. After that, 26 g of KMnO_4_ was very slowly added to the solution under vigorous stirring. The temperature was kept below 20 °C during mixing, then at 30 ± 5 °C for 2 h and at room temperature overnight, always under stirring. Then, 240 mL of deionized water was slowly added to prevent the temperature from rising above 70 °C. Subsequently, the solution was stirred for 60 min and diluted with 600 mL of deionized water. Then, 26 mL of H_2_O_2_ (33%) was added dropwise, turning the solution to yellowish-brown. This solution is finally sonicated in an ultrasounds bath for 60 min to achieve the exfoliation of the oxidized material. This GO suspension contains GONFs (40% of the initial weight of CNFs), completely exfoliated materials like few-layer graphene oxide flakes (FLGOs) and GO quantum dots (GOQDs) and other inorganic salts and thus, separation and posterior washing of phases were necessary. Product was washed by centrifugation at 9500 rpm with HCl (10%) first and deionized water until neutral pH. The clean precipitate was dispersed in deionized water and separated from the rest of the GO products (FLGOs and GOQDs) as the precipitate of its centrifugation at 4500 rpm. More details about the whole process by differential degressive centrifugation can be found elsewhere [29]. Finally, fractions were reduced in suspension by the hydrothermal reduction method [37] in a 40 mL autoclave placed in the oven at 140, 180 and 220 °C for 6 h. In this case, 1 g of GONFs (obtained after drying the GONF solution at 60 °C in a vacuum oven), was dispersed in 25 mL of deionized water. After reduction, products were dried at 60 °C in a vacuum oven overnight. Reduced materials will be indicated hereafter as RGONF-140, RGONF-180, and RGONF-220.

### 2.2. Physicochemical Characterization

Characterization of GONF and RGONF samples was carried out by X-ray diffraction (XRD), X-ray photoelectron spectroscopy (XPS), elemental analysis (EA), and N_2_ and CO_2_ physisorption at 77 K and 273 K, respectively. GONF dispersions were dried at 60 °C overnight for its characterization.

XRD patterns of GONFs and RGONFs were acquired in a Bruker D8 Advance Series 2 diffractometer (Bruker AXS, Karlsruhe, Deutschland). The angle range scanned was 3–55° using a counting step of 0.02° and a counting time per step of 4 s. XRD data were fitted using the structure analysis software TOPAS (Bruker AXS, Karlsruhe, Deutschland). The mean interlayer spacings (*d*-spacing) were evaluated from the position of the corresponding peak applying Bragg’s Law [38], while the mean crystallite sizes along c axis (*L*_c_) were calculated using the Scherrer formula, with a value of K = 0.89 [38]. From these, the number of graphene layers (*n*) was calculated as (*L*_c_/*d*_002_) + 1.

The bulk and surface chemistry was analysed by EA and XPS, respectively. The ESCAPlus OMICROM spectrometer (Omicron, Houston, TX, USA), equipped with a hemispherical electron energy analyser, was operated at 18.75 kV and 12 mA (225 W), using a monochromatic AlKα X-ray source (*hv* = 1486.7 eV) and under vacuum (<5 × 10^−9^ Torr). A survey scan between 1000 and 0 eV was acquired using steps of 0.5 eV and 200 ms of dwell, while C 1s region was acquired each 0.1 eV for 500 ms. Analyser pass energies of 50 and 20 eV were used for survey scans and 20 eV for C1s region, respectively. Elemental analysis of GONFs and RGONFs was performed in a CHNS-O Analyser Thermo FlashEA 1112 (Thermo Fisher Scientific, Waltham, MA, USA).

The textural properties were measured using a Micromeritics ASAP2020 apparatus for N_2_ and CO_2_ physisorption at 77 and 273 K, respectively. The specific surface area (*S*_BET_) was calculated by the BET method applied to the N_2_ adsorption isotherm. The total pore volume (*V*_t_) was calculated from the N_2_ adsorbed volume at a relative pressure of *p*/*p*_0_ > 0.994. In addition, micropore surface area (*S*_mic_) and the total micropore volume (*V*_t_mic_) were calculated by the Dubinin–Radushkevich equation and the adsorbed volume at *p*/*p*_0_ > 0.031 (pores < 0.8 nm), respectively, using the CO_2_ adsorption data.

The morphology of carbon nanofilaments was visualized by transmission electron microscopy (TEM; Tecnai F30, FEI company, Eindhoven, The Netherlands).

### 2.3. Electrochemical Characterization

Electrochemical measurements were carried out in a three-electrode electrochemical cell and an Autolab PGSTAT302 (Metrohm, Utrecht, Netherlands) potentiostat–galvanostat was used to record the data. A carbon rod and a reversible hydrogen electrode (RHE) were used as counter and reference electrodes, respectively. All potentials in the text are referred to the RHE. Working electrodes were prepared by depositing a layer of a carbon ink on glassy carbon (diameter = 7 mm), resulting in a mass loading of the active material of 1 mg·cm^−2^. Carbon inks were obtained by dispersing 2 mg of the corresponding material in a deionized water/isopropyl alcohol 50/50 (*v*/*v*) solution containing 15 wt. % Nafion^®^ (Sigma Aldrich, 5 wt. %). The inks were sonicated for 30 min.

Working electrodes were introduced in the base electrolyte (0.5 M H_2_SO_4_) saturated with nitrogen. Cyclic voltammetries were performed from 0.2 to 0.8 V vs. RHE at several scan rates: 5, 10, 20, 50, 100, and 200 mV·s^−1^. Specific capacitances, *C* (F·g^−1^), were obtained by integration of the area enclosed in the cyclic voltammograms (CV), according to Equation (1) [39,40]:

(1) $$C\;\left[F\cdot g^{-1}\right]=\frac{\oint I\;dV}{2\;m\;\nu \;∆V}$$

where *I* is the current (A), *V* is the potential (V), ∆*V* is the potential window (V), *ν* is the scan rate (V·s^−1^), and *m* is the mass of the active material in the working electrode (g).

A frequency response analyser (FRA) was employed to record the electrochemical impedance spectroscopy (EIS) measurements. A frequency range from 100 kHz to 0.01 Hz was always used with a 10 mV r.m.s. AC amplitude around 0.5 V vs RHE.

## 3. Results and Discussion

### 3.1. Changes in the Physicochemical Properties of GONFs after Reduction

**Figure 1:**
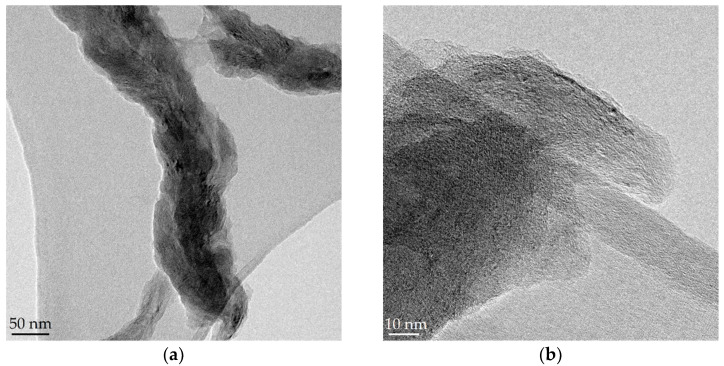

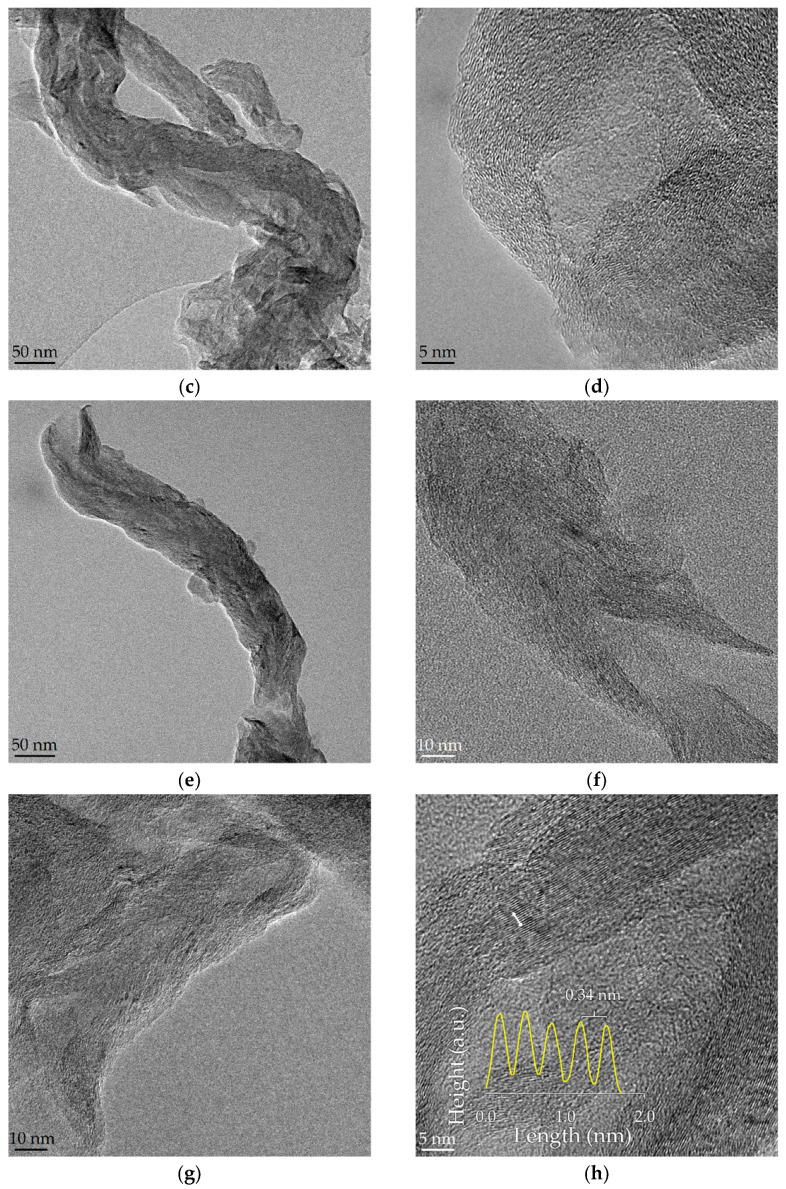
TEM images of: (**a**,**b**) graphene oxide nanofibers (GONF); and reduced GONFs (RGONFs) obtained at (**c**,**d**) 140, (**e**,**f**) 180, and (**g**,**h**) 220 °C. Height profile of graphene layers in a turbostratic stack is included in (**h**).

GONFs, characterized by preserving the filamentous structure of the CNFs used in the synthesis but with a higher degree of interlayer spacing (*d* > 0.75 nm), presented a higher electrochemical capacitance than the bare CNFs [28]. In this work, GONFs were used as the starting material to study the effect of the hydrothermal reduction temperature (140, 180, and 220 °C) on the physicochemical and electrochemical properties of RGONFs. The 1-D morphology of GONF and RGONFs was revealed by TEM as images in Figure 1 shown. These structures presented fishbone arrangement, inherited from the original CNFs, where the graphite/graphene stacks form an oblique angle to the longitudinal growth axis [41]. GONF (Figure 1a,b) showed disordered arrangements of graphene layers due to the intercalated basal oxygenated groups (hydroxyl and epoxide). In this case, a non-oxidized core preserves the main structure of the nanofilament. As a result of the hydrothermal reduction, RGONFs (Figure 1c–h) presented more compact structures and better definition of layers and edges. Regarding the effect of the temperature used in the hydrothermal reduction, the higher the temperature, the greater the restacking of the graphene layers. In Figure 1h, a height profile of graphene layers in a turbostratic stack of the RGONF-220 sample is shown, with a regular interlayer distance of 0.34 nm.

**Figure 2:**
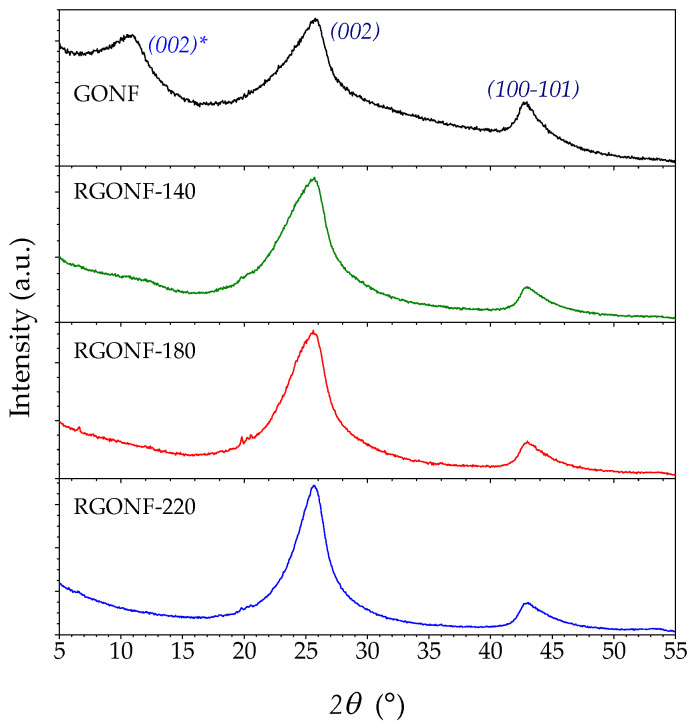
X-ray diffraction (XRD) patterns of GONF and RGONFs obtained at 140, 180, and 220 °C.

XRD patterns of GONF and RGONFs and their associated structural parameters such as *d*-spacings, *L*_c_ and the number of layers are depicted in Figure 2 and listed in Table 1, respectively. GONF showed the presence of two main diffraction peaks corresponding to the basal plane (002) of the graphite stacking at 25.9° (2*θ*) and the shifted (002) plane (labelled with *) at lower angles (around 10.9°), corresponding to *d*-spacings of 0.344 and 0.810 nm, respectively. A crystal arrangement of 0.81 nm corresponds to the distance between layers with oxygen intercalated graphite/graphene domains that conform to this fishbone-type nanofilament and is in line with typical values for GO materials [42]. The presence of (002) and (002)* planes is a consequence of the partial oxidation that takes place from the outside towards the inner axis of the starting CNF and is essential in GONF materials as they maintain their tubular structure [29]. After the reduction process, the (002) plane (25.6–25.8°) is recovered at the expense of the (002)* plane motivated by the graphene layers approaching after the removal of oxygenated groups [43]. Only RGONF-140 showed a residual broad band of (002)*. All RGONF samples presented a slightly higher interlayer spacing (about 0.345–0.347 nm) than that in theoretical graphite (0.3354 nm), as confirmed by XRD and in line with TEM images, as is consistent with a non-graphitic or turbostratic arrangement [44,45]. In fact, a deconvolution of this asymmetric peak permits differentiate between turbostratic and hexagonal graphite and to qualitatively calculate the graphitization degree achieved [43,45,46]. At 220 °C, the average number of graphene layers (*L*_c_/*d*_002_ + 1) was increased from 7.0 in GONF (*L*_c_ = 2.1 nm) to 9.5 layers in RGONF-220 (*L*_c_ = 2.9 nm). These slightly thicker graphite domains are due to the light restacking of the graphene layers by strong π–π interactions and Van der Waals forces between graphene layers and intercalated water molecules [32,42,47,48].

**Figure 3:**
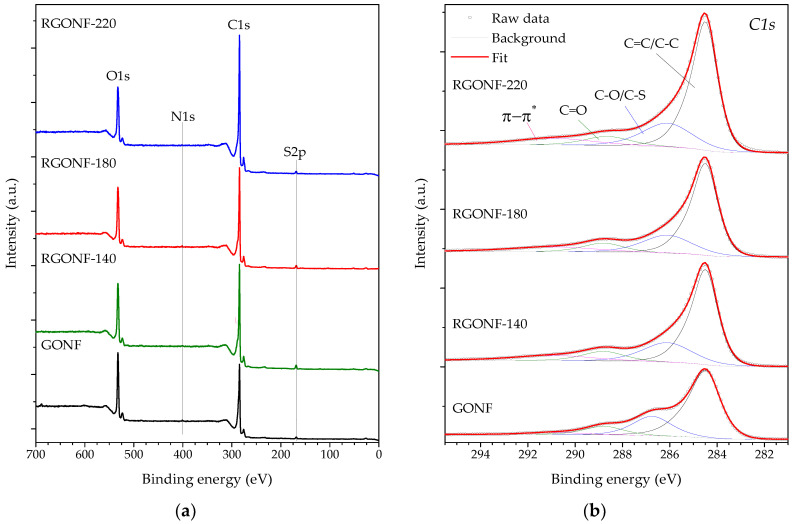
(**a**) X-ray photoelectron spectroscopy (XPS) Survey; and spectra of the (**b**) C1s region of GONF and RGONFs obtained at 140, 180, and 220 °C.

The bulk and surface compositions of GONF and RGONFs were analysed by elemental analysis and XPS, respectively, as presented in Figure 3 and Table 2. XPS survey spectra of the samples (Figure 3a) showed an inverse behaviour in the trends of C and O which increased and decreased, respectively, as the reduction temperature increased. Surface O content decreased from 18.4 at. % in GONF to a minimum of 12.4 in RGONF-220 (or 32.5 and 17.8 wt. % for the bulk composition, respectively). C/O atomic ratios of RGONFs ranged from 5.3 and 5.4 for RGONF-140 and RGONF-180 to 7.0 for RGONF-220, and they were within typical values for reduced GO obtained by hydrothermal processes [49,50,51]. N and S contents are derived from the use of NaNO_3_ and H_2_SO_4_ during GONF synthesis. The evolution of the different oxygen-containing groups was followed by deconvolution of the C 1s high-resolution regions (Figure 3b and Table 2). Details about C 1s deconvolution can be found elsewhere [9,29]. C 1s was fitted to the components: sp^2^ (C=C) and sp^3^ hybridized carbon (C–C), C–O bonds in hydroxyls and epoxides (in-plane)s and C–S in sulfonic acid and organosulfates [9], C=O bonds in carbonyls and carboxyls (edge groups), and the π–π* shake-up satellite belonging to aromatic and unsaturated bonds. All samples showed both in-plane and edge oxygen functionalities disrupting the sp^2^ graphene network. Reduction removed both, although only RGONF-220 reached a significant variation for edge functional groups. More evident was the restoration of the saturation in RGONFs as incremented the contribution of π–π* transitions.

Although the hydrothermal reduction only causes the partial oxidation of the starting GONFs, it is enough to modify some physicochemical properties, such as layer restacking or texture.

**Figure 4:**
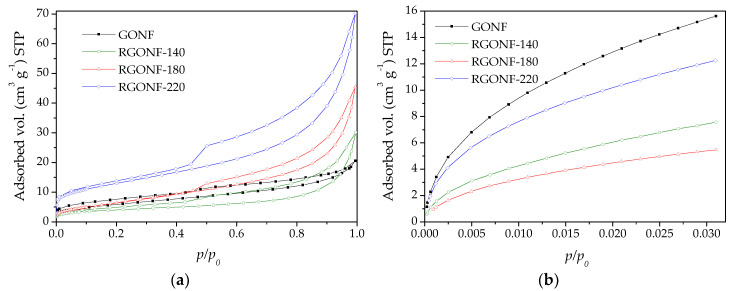
(**a**) Isotherms of N_2_ at 77 K and (**b**) CO_2_ at 273 K for GONF and RGONFs.

Figure 4 shows the N_2_ and CO_2_ isotherms of GONF and RGONF samples. Likewise, textural parameters are listed in Table 3. Based on the results of N_2_ physisorption, GONF and RGONFs exhibited type IV isotherms (according to the IUPAC [52]), typical of mesoporous solids, with a H3 hysteresis loop closing at *p*/*p*_0_ = 0.45. This hysteresis type corresponds to non-rigid aggregates of plate-like particles, where condensation takes place between parallel plates or open slit-shaped capillaries [52]. The sharp step-down located in desorption branch at *p*/*p*_0_ = 0.43–0.53 is attributed to pore blocking in pore necks [52]. BET surface areas (*S*_BET_ in Table 3) obtained using the adsorption branch of the N_2_ physisorption decreased from 21.7 m^2^·g^−1^ for GONF to 14.3 m^2^·g^−1^ after hydrothermal reduction at the lowest tested temperature (140 °C). Higher temperatures resulted in an increase of *S*_BET_ (25.1 and 46.3 m^2^·g^−1^ for RGONF-180 and RGONF-220, respectively). Oxygen and sulphur functional groups contained in GONFs hinder the access for N_2_ to certain type of pores. After reduction, these groups are partially removed and the graphene layers are approached. Consequently, the development of porosity in RGONFs is mainly due to the mesopores generated in their more difficult compaction as *V_t_* values indicated. RGONF structures, where the layer restacking occurred, are more rigid than GONFs. On the other hand, due to the importance of micropores in GO samples, which are ascribed to the cuneiform pores between the graphenes (slit type pores), CO_2_ adsorption measurements were also carried out in order to know the surface area of GONF and RGONFs including narrow micropores (from 0.4 nm). The micropore surface areas (*S*_mic_) offered the same evolution as observed for *S*_BET_ but showing values much higher than those. In this case, the progressive removal of surface functional groups resulted in the clear recovery of the micropore surface area. An even higher development of micro- and mesoporosity could be hydrothermally obtained at more severe conditions [43].

### 3.2. Electrochemical Characterization of GONF and RGONFs

**Figure 5:**
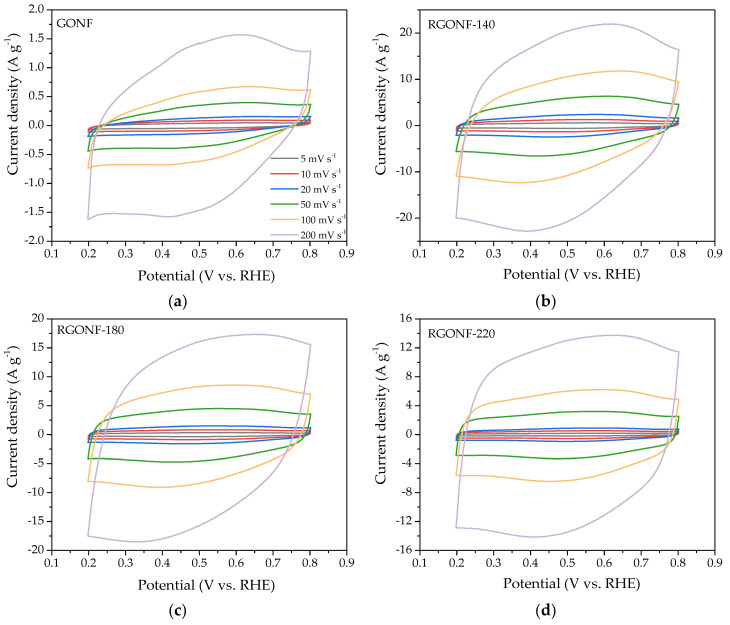
Cyclic voltammograms (CV) from 0.2 to 0.8 V vs. reversible hydrogen electrode (RHE) at several scan rates (5, 10, 20, 50, 100, and 200 mV·s^−1^) of (**a**) GONF; and RGONFs obtained at (**b**) 140; (**c**) 180; and (**d**) 220 °C. Aqueous electrolyte: 0.5 M H_2_SO_4_.

Cyclic voltammograms (CV) were recorded in deaerated 0.5 M H_2_SO_4_ at six different scan rates: 5, 10, 20, 50, 100, and 200 mV·s^−1^ for GONF and RGONFs. Figure 5 shows the current density-potential behavior for every carbon material at the different scan rates. As expected, the increase of scan rate is associated with an increase of the charge. A quasi rectangular shape is observed for all of them, accounting for the influence of double layer capacitance, together with a small redox contribution with peak current density at about 0.6 V vs. RHE in the positive-going scan and 0.4 V vs. RHE in the negative-going one.

**Figure 6:**
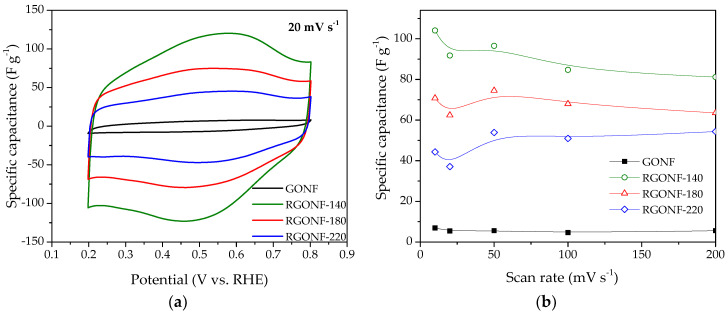
(**a**) Specific capacitance vs. potential curves at 20 mV·s^−1^ of GONF and RGONF samples. (**b**) Specific capacitance as a function of the scan rate. Aqueous electrolyte: 0.5 M H_2_SO_4_.

Specific capacitances were obtained following the Equation (1). Figure 6a shows an example of the specific capacitance-potential curves obtained at 20 mV·s^−1^ for GONF and RGONFs, while the capacitance values as function of the scan rate are given in Figure 6b. In spite of the partial removal of polar oxygen groups upon hydrothermal reduction treatments (oxygen groups may enhance the hydrophilicity and thus the available electrochemical surface area), an important increase of the double-layer current (see Figure 6a) was observed for RGONFs with specific capacitances from 8- to 16-times higher than that obtained for GONF (~6 F·g^−1^). RGONFs exhibit a much higher capacitance than GONF, with a good capacitance retention (Figure 6b), despite the relatively low surface area of RGONFs, as evidenced by N_2_ and CO_2_ physisorption experiments. Although the morphology and structure of RGONFs are different to those reported in the literature regarding reduced graphene oxide, carbon nanofibers decorated with reduced graphene oxide or hybrid materials [53,54,55,56,57], other graphene-based materials have exhibited similar values of specific capacitances in the order of 100 F·g^−1^ with BET surface areas of same order of magnitude than RGONFs reported in this work [21,58].

The samples reduced at 140, 180, and 220 °C present capacitance values up to 104, 75, and 55 F·g^−1^, respectively. RGONFs present fishbone arrangement (from the original CNFs) with turbostratic stacks forming an oblique angle to the longitudinal growth axis (Figure 1). These highly crystalline structures consisting on few-atom-thick stacks with sp^2^-hybridized carbon and lateral dimensions less than 100 nm are expected to exhibit unique properties due to edge effects, quantum confinement and structural defects [58,59]. Consequently, these structures may confine energy band gaps and delocalized charge carriers, resulting in high specific capacitance. On the other hand, other authors have reported an enhanced capacitance of reduced graphene oxide in comparison to graphene oxide [60,61]. The latter can be related to: (i) an increase of the electrical conductivity upon hydrothermal reduction, which favours the electron mobility at RGONF electrodes [61,62,63], and/or (ii) the particular porous texture and turbostratic structure of RGONFs, which is less restrictive for ion/electrolyte transport. In this context, an important recovery of the π–π* shake-up satellite contribution was observed by XPS (Table 2) upon hydrothermal reduction, as well as a progressive removal of oxygenated species, which may lead to an improved carbon conductivity [7]. On the other hand, a significant decrease of the narrow microporosity was observed after reduction treatments (Table 3, CO_2_ physisorption). These narrow micropores may hinder the access of the ions to the surface of carbon electrodes. Additionally, reduction of GONFs is expected to reduce the band gap and to increase the electron-hole pairs, which leads to an enhancement of the quantum confinement [64].

Interestingly, as the reduction temperature decreases, a larger electrochemical capacitance was obtained. These results indicate that the material reduced at 140 °C presents balanced characteristics for charge storage. Higher temperatures resulted in the increase of narrow microporosity as well as some slight restacking of the graphene layers (as confirmed by XRD), which may result in a more restricted quantum confinement effect [32]. Whereas the bare GONF exhibits poor electrical conductivity hindering the electron mobility. Additionally, RGONF-140 presented the highest surface S content (XPS, Table 2) and the largest O concentration among RGONFs. Sulphur and oxygen species have also been reported to present a positive effect on the capacitance of carbon materials in both acid and alkaline electrochemical environments, as a result of Faradaic reactions (pseudocapacitive contribution) and/or a lower affinity to adsorb water by changes in the charge distribution on carbon atoms [34,65,66,67,68].

In order to gain further insights on the relative influence of pseudocapacitive contribution to the total specific capacitance, current density was deconvoluted in two components as in Equation (2), as reported in [69]:

*I*(*ν*) = *k*_1_*ν* + *k*_2_*ν*^1/2^ (2)

where *I* is the current (A), *k*_1_ and *k*_2_ are scan rate independent constants and *ν* is the scan rate (V·s^−1^). The component associated to *k*_1_ (proportional to *ν*) encompasses electrochemical double layer charging (non faradaic), while *k*_2_ (proportional to *ν*^1/2^) is related to diffusion limited charge from the faradaic contribution of pseudocapacitance, as derived from the Randles–Sevcik equation [39,70,71]. By linearization of Equation (2), a simple linear regression can be applied to the representation of *I*/*ν*^1/2^ against *ν*^1/2^ as follows in Equation (3):

*I*(*ν*)/*ν*^1/2^ = *k*_1_*ν*^1/2^ + *k*_2_ (3)

**Figure 7:**
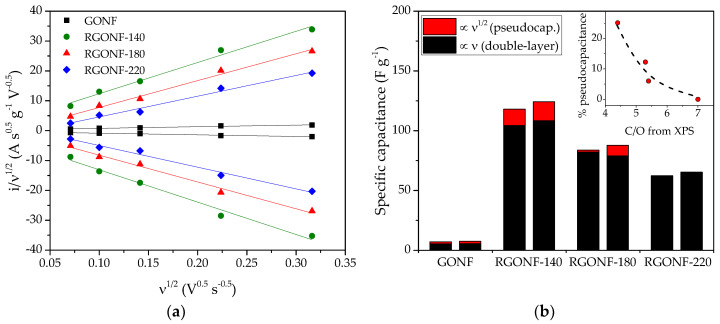
(**a**) Dependence of *i*/*ν*^1/2^ with *ν*^1/2^ for GONF and RGONFs at 0.5 V vs. RHE. (**b**) Specific capacitances determined from the deconvolution in Figure 7a separated in pseudocapacitance (red, ∝ *ν*^1/2^) and double layer capacitance (black, ∝ *ν*), for the positive- and negative-going scans being the left and right column, respectively, for each material; the inset represents the percentage of pseudocapacitance as a function of C/O ratio from XPS.

Figure 7a depicts the variation of *i*/*ν*^1/2^ with the square root of ν for all the materials, including both positive- and negative-going values of current at 0.5 V vs. RHE, together with their linear regression. In RGONF materials, the intercept with the y-axis increases as reduction temperature decreases, indicating a larger pseudocapacitive contribution in the nanofibers treated at lower temperature. Double layer capacitances and pseudocapacitances were calculated from these linear regressions for all the samples, as represented in Figure 7b. The double layer contribution to the total specific capacitance accounts for 87–100% according to this methodology. The largest pseudocapacitance was observed for RGONF-140 (Figure 7b), which is related to the larger extent of O and S groups on the structure of the former material in comparison with the other two RGONFs. The relative contribution of redox processes to the total capacitance is depicted in the inset graph of Figure 7b as a function of the C/O ratio, determined by XPS. As expected, an increase of oxygen surface groups (decrease of C/O ratio) in the form of quinone/hydroquinone (C=O species, XPS, Table 3) results in an increase of the relative pseudocapacitive effect.

**Figure 8:**
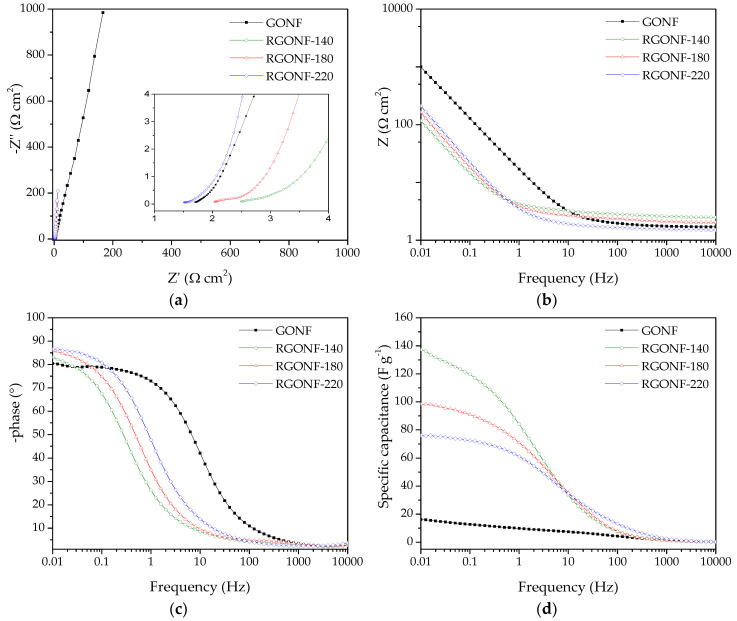
(**a**) Nyquist impedance plots with a 10 mV AC amplitude around 0.5 V of GONF and RGONFs. (**b**) Frequency-dependent impedance modulus Bode plots with a 10 mV AC amplitude around 0.5 V. (**c**) Frequency-dependent phase angle Bode plots with a 10 mV AC amplitude around 0.5 V. (**d**) Specific capacitance vs. frequency curves with a 10 mV AC amplitude around 0.5 V of GONF and RGONFs. Aqueous electrolyte: 0.5 M H_2_SO_4_.

In order to study the overall resistance and the electrolyte ion transport, alternative current EIS measurements were carried out at 0.5 V vs. RHE with 10 mV amplitude (r.m.s.). Figure 8a shows the Nyquist plots, while the Bode-impedance and Bode-phase plots are given in Figure 8b,c, respectively, for all the materials. The Nyquist plots of RGONFs exhibit a sharp increase of the imaginary component of impedance (*Z*”) with the decrease of frequency, which is indicative of their almost ideal capacitive behaviour, together with a small semicircle at high frequencies [55]. Interestingly, RGONFs present a higher slope at low frequencies than GONF indicating faster ion movement [53,72]. Therefore, RGONF materials present a low charge-transfer resistance with high electrolyte diffusion. The intersection point with the real axis in the Nyquist plot corresponds to the equivalent cell series resistance (*R_s_*), which is in the range 1.5–2.4 Ω·cm^−2^. The latter is also evident in the Bode-impedance plot at high frequencies (Figure 8b).

Figure 8d shows the dependence of the specific capacitance (F·g^−1^) with the frequency, where capacitance values were obtained by Equation (4):

(4) $$C=-\left(\frac{1}{2\pi fZ^{\prime \prime }}\right)$$

**Figure 9:**
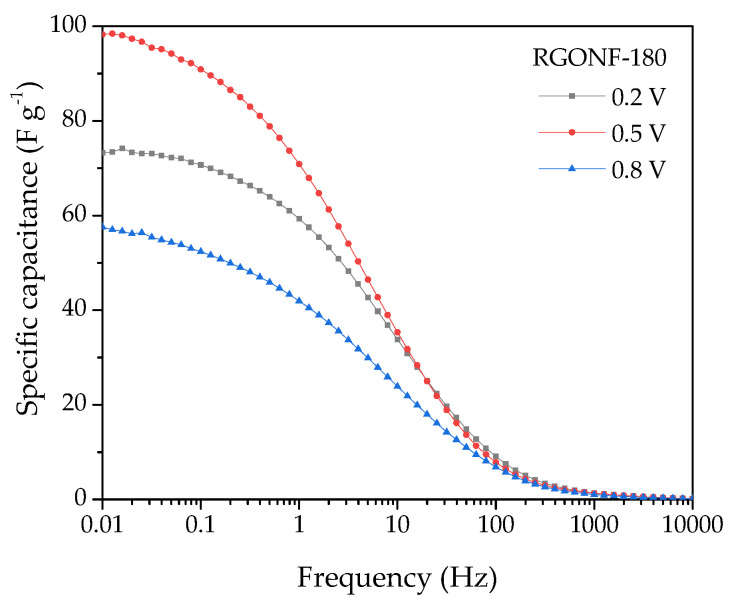
Specific capacitance vs. frequency curves with a 10 mV AC amplitude around 0.2, 0.5, and 0.8 V of RGONF-180. Aqueous electrolyte: 0.5 M H_2_SO_4_.

As expected, an increase of the capacitance as the frequency decreases is evident for all materials. At the lowest frequencies (0.01 Hz), the curves reach a plateau at the maximum capacitances. In line with the results obtained by CV, improved capacitances were obtained for RGONFs (76–137 F·g^−1^) in comparison to GONF (16 F·g^−1^). On the other hand, RGONF-140 presented the highest capacitive performance (137 F·g^−1^). As the severity of the reduction conditions increases, a lower capacitance was obtained for RGONF-180 (98 F·g^−1^) and RGONF-220 (76 F·g^−1^), following the same trend already observed by CV experiments. Although superior capacitance values were obtained by EIS measurements in comparison to those calculated by CV, comparable results were observed at 0.5 V vs. RHE (see Figure 8d and Figure 7b). On the other hand, a decrease of the capacitance determined by EIS measurements was evident at 0.2 and 0.8 V vs. RHE. For example, values of 72 and 58 F·g^−1^ were recorded at 0.2 and 0.8 V vs. RHE for the carbon RGONF-180 (Figure 9), whereas a higher capacitance (98 F·g^−1^) was observed at 0.5 V. This is ascribed to the contribution of the pseudocapacitive component observed in CV experiments at potentials close to 0.5 V vs. RHE, and related to reversible redox processes from oxygen surface groups, most probably quinone-hydroquinone redox pair.

This work reveals that hydrothermal reduction of GONF leads to an enhancement of the specific capacitance, with reduction temperature playing an important role in the morphology and structure of the RGONF materials and also on the electrochemical performance. These results are of interest to design electrodes with potential applications in electrochemical energy conversion and storage, such as supercapacitors, batteries or fuel cells. However, further electrochemical characterization would be necessary to achieve the requirements of commercial devices. For instance, optimization of the mass loading and thickness of the electrode and long-term stability studies.

## 4. Conclusions

In this work, the influence of hydrothermal reduction at different temperatures (140, 180, and 220 °C) of fishbone graphene oxide nanofibers was carried out, obtaining a novel material with enhanced electrochemical capacitance. After reduction, the resultant carbon presented the partial removal of basal functional groups on graphene/graphite domains, resulting in accessible turbostratic domains and restored electronic conductivity. In particular, RGONFs showed an improved double-layer current with capacitance values from 8- to 16-times higher than GONF, which is ascribed to the unique structure of RGONFs. Hydrothermal reduction at 140 °C led to the highest capacitance as evidenced by cyclic voltammetry and electrochemical impedance spectroscopy (up to 137 F·g^−1^). A rise in the hydrothermal reduction temperature resulted in a removal of S- and O-containing functional groups and an increase of microporosity, leading consequently to a lower specific capacitance. Additionally, XRD evidenced the presence of slightly thicker graphite domains for the materials treated at 180 and 220 °C, which might decrease the quantum confinement effect, reducing capacitance values.

The proposed approach represents a novel strategy for the production of reduced graphene oxide nanofibers with tuneable properties. The reduced materials exhibit a unique structure and morphology with an enhanced capacitance in comparison to GONFs.

## Figures and Tables

**Table 1:** Structural parameters of GONF and RGONFs obtained at 140, 180, and 220 °C determined by XRD.

Sample	*d* (nm)		*L*_c_ (nm)		*n*	
	(002) *	(002)	(002) *	(002)	(002) *	(002)
GONF	0.810	0.344	1.5	2.1	2.9	7.0
RGONF-140	-	0.347	-	2.1	-	7.0
RGONF-180	-	0.345	-	2.3	-	7.6
RGONF-220	-	0.346	-	2.9	-	9.5

* For shifted (002) peak.

**Table 2:** Bulk and surface composition measured by elemental analysis and XPS, respectively.

Sample	EA (wt. %)					XPS—Survey (at. %)					XPS—C 1s (%)			
	C	O	N	S	H	C	O	N	S	C/O	C sp^2^	C–O	C=O	π–π*
GONF	55.9	32.5	0.1	1.6	2.4	80.1	18.4	0.3	1.2	4.4	62.2	23.7	10.4	3.7
RGONF-140	67.4	22.9	0.1	1.8	1.3	82.3	15.5	0.3	1.9	5.3	56.4	23.7	9.8	10.0
RGONF-180	67.7	21.9	0.1	1.9	1.3	82.9	15.4	0.3	1.4	5.4	56.8	23.0	9.4	10.8
RGONF-220	74.2	17.8	0.1	1.1	1.2	86.4	12.4	0.3	1.0	7.0	58.9	22.9	8.0	10.1

**Table 3:** Textural parameters of GONF and RGONFs obtained at 140, 180, and 220 °C.

Sample	N_2_		CO_2_	
	*S*_BET_ (m^2^·g^−1^)	*V*_t_^a^ (cm³·g^−1^)	*S*_mic_^b^ (m^2^·g^−1^)	*V*_t_mic_^c^ (cm³·g^−1^)
GONF	21.7	0.032	101.8	0.029
RGONF-140	14.3	0.047	43.6	0.014
RGONF-180	25.1	0.070	36.4	0.010
RGONF-220	46.3	0.109	79.5	0.022

^a^ Total pore volume at *p*/*p*_0_ = 0.994 (maximum pore size of 495 nm); ^b^ *S*_mic_ using the Dubinin–Radushkevich equation; ^c^ Total pore volume at *p*/*p*_0_ = 0.031 (maximum pore size of 0.8 nm).
